# The New Is Old: Novel Germination Strategy Evolved From Standing Genetic Variation in Weedy Rice

**DOI:** 10.3389/fpls.2021.699464

**Published:** 2021-06-21

**Authors:** Chengchuan Zhou, Yang Feng, Gengyun Li, Mengli Wang, Jinjing Jian, Yuguo Wang, Wenju Zhang, Zhiping Song, Linfeng Li, Baorong Lu, Ji Yang

**Affiliations:** ^1^National Observations and Research Station for Wetland Ecosystems of the Yangtze Estuary, Ministry of Education Key Laboratory for Biodiversity Science and Ecological Engineering, Fudan University, Shanghai, China; ^2^Shanghai Key Laboratory of Plant Functional Genomics and Resources, Shanghai Chenshan Botanical Garden, Shanghai, China

**Keywords:** weedy rice, crop feralization, germination strategy, rapid adaptation, standing genetic variation

## Abstract

Feralization of crop plants has aroused an increasing interest in recent years, not only for the reduced yield and quality of crop production caused by feral plants but also for the rapid evolution of novel traits that facilitate the evolution and persistence of weedy forms. Weedy rice (*Oryza sativa* f. *spontanea*) is a conspecific weed of cultivated rice, with separate and independent origins. The weedy rice distributed in eastern and northeastern China did not diverge from their cultivated ancestors by reverting to the pre-domestication trait of seed dormancy during feralization. Instead, they developed a temperature-sensing mechanism to control the timing of seed germination. Subsequent divergence in the minimum critical temperature for germination has been detected between northeastern and eastern populations. An integrative analysis was conducted using combinations of phenotypic, genomic and transcriptomic data to investigate the genetic mechanism underlying local adaptation and feralization. A dozen genes were identified, which showed extreme allele frequency differences between eastern and northeastern populations, and high correlations between allele-specific gene expression and feral phenotypes. Trancing the origin of potential adaptive alleles based on genomic sequences revealed the presence of most selected alleles in wild and cultivated rice genomes, indicating that weedy rice drew upon pre-existing, “conditionally neutral” alleles to respond to the feral selection regimes. The cryptic phenotype was exposed by activating formerly silent alleles to facilitate the transition from cultivation to wild existence, promoting the evolution and persistence of weedy forms.

## Introduction

Feralization of crop plants has aroused an increasing interest in recent years, not only for the reduced yield and quality of crop production caused by feral plants but also for the rapid evolution of novel traits that facilitate the persistence of feral populations in natural or semi-natural habitats (Gressel, 2005; Vigueira et al., 2013). Feral plants often partially or totally lose traits associated with domestication and re-acquire wild-like traits, such as freely-dispersing seed and strong seed dormancy. This process is, however, not a mere reversal of domestication (Gering et al., 2019). The wild-like traits may reemerge through different genetic mechanisms in the polyphyletic feral plants that have independently evolved from different populations or varieties of the same cultivated progenitor in ecologically similar yet geographically distant environments (Wu et al., 2021). The feral plants and their cultivated relatives thus provide appealing systems for studying convergent or parallel evolution. Understanding the genetic basis underlying the key traits associated with weed success will not only gain insight into the molecular mechanisms underpinning feralization, but also provide clues to devising more effective weed control strategies and breeding better crops.

Weedy rice (*Oryza sativa* f. *spontanea*), also referred as red rice, is a conspecific weed of cultivated rice (*O. sativa*), which has caused significant reduction in rice grain yield and quality worldwide (Delouche et al., 2007). Weedy rice can originate either directly from cultivated rice via de-domestication (endoferality) or by hybridization between cultivated rice and its wild relatives (exoferality) (Ellstrand et al., 2010). Independent and recurrent origins of weedy rice have been reported in many rice-planting regions around the world, though some weed-adaptive traits, such as seed shattering, strong seed dormancy and pericarp pigmentation, are shared by most independently-evolved weedy rice strains (Cao et al., 2006; Huang et al., 2017; Vigueira et al., 2019; Hoyos et al., 2020). Evolutionary genomic studies of weedy rice have revealed a large number of gene loci associated with weed adaptation. However, little overlap occurred between the weed-adaptive loci and those related to domestication, and between the loci that have contributed to rice feralization in different regions (Li et al., 2017; Qiu et al., 2017, 2020). The re-occurrence of wild type traits in weedy rice are thus not based on simple back mutations from the allele associated with domesticated traits to the wild-type allele of the wild progenitor at domestication-related loci, and the shared weedy traits of distinct weedy rice strains most likely occur through different genetic mechanisms (Qi et al., 2015; Qiu et al., 2020).

The weedy rice strains distributed in eastern and northeastern China are considered to have an endoferal origin since they exist outside the range of the wild progenitor of rice. Genomic sequencing and genetic diversity analyses have shown that the weedy rice populations from northeastern China are strongly associated with *japonica* rice cultivars and those from eastern China with *indica* rice cultivars, suggesting the independent feralization events occurred with distinct cultivated progenitors in different regions (Cao et al., 2006; Qiu et al., 2017). In addition to genetic divergence, morphological variations in hull color, awn length and color, grain length/width ratio, etc., have also been reported within and among populations (Zhang et al., 2017). Nevertheless, a notable feature that is shared by the weedy rice from eastern and northeastern China is that their seeds do not have primary dormancy. Instead, these plants have developed a temperature-sensing mechanism to control the timing of seed germination during feralization (Xia et al., 2011). This trait is not present either in cultivated or in wild rice; thus, its evolution is essentially not a case of de-domestication. Meanwhile, divergence in the minimum critical temperature for germination has been detected between the populations from the northeastern region and those from the eastern region, suggesting rapid adaptive evolution in germination behavior to local climatic conditions (Xia et al., 2011).

It has been well-recognized that, during the domestication process, crops usually experience genetic bottlenecks (Doebley et al., 2006; Burke et al., 2007). The small initial population size and the intense selection pressure for agronomic traits can lead to dramatic reduction in genetic diversity (Eyre-Walker et al., 1998; Allaby et al., 2017). It is now known that, similar to domestication, there exist severe genetic bottlenecks during the de-domestication process, as shown in weedy rice (Reagon et al., 2010; Qiu et al., 2017). The decrease in genetic diversity subsequent to bottleneck events and the deficient heterozygote associated with the predominant selfing nature of weedy rice can extensively reduce its fitness and evolutionary potential. Yet, rather than suffering from the detrimental effects of low genetic variation, weedy rice has rapidly evolved a novel germination strategy in the past few decades to adapt to temperature changes with latitude. It seems paradoxical: How does weedy rice overcome the hazards of genetic homogeneity that might have been caused by the bottleneck event, selfing and genetically homogeneous cultivated progenitors, to persist and evolve rapidly at different geographical regions under contrasting feral selection regimes?

To understand the genetic basis of adaptive divergence in weedy rice germination, whole-genome resequencing of weedy rice from different populations was first conducted in this study to detect the region-specific SNPs. Then, the SNP data was integrated with the gene expression profiles of weedy rice at different germination stages to identify genes that were differentially expressed and possessed distinct alleles between the samples from different regions. We hypothesize that the differentially expressed genes harboring region-specific SNPs are more likely to be associated with adaptive diversification in germination. The allele frequencies of potential beneficial SNPs were further validated by targeted SNP genotyping. Our results suggested that the feral selection regimes might have driven the fixation of pre-existing, “conditionally neutral” alleles in weedy rice.

## Materials and Methods

### Weedy Rice Sampling

**Figure 1:**
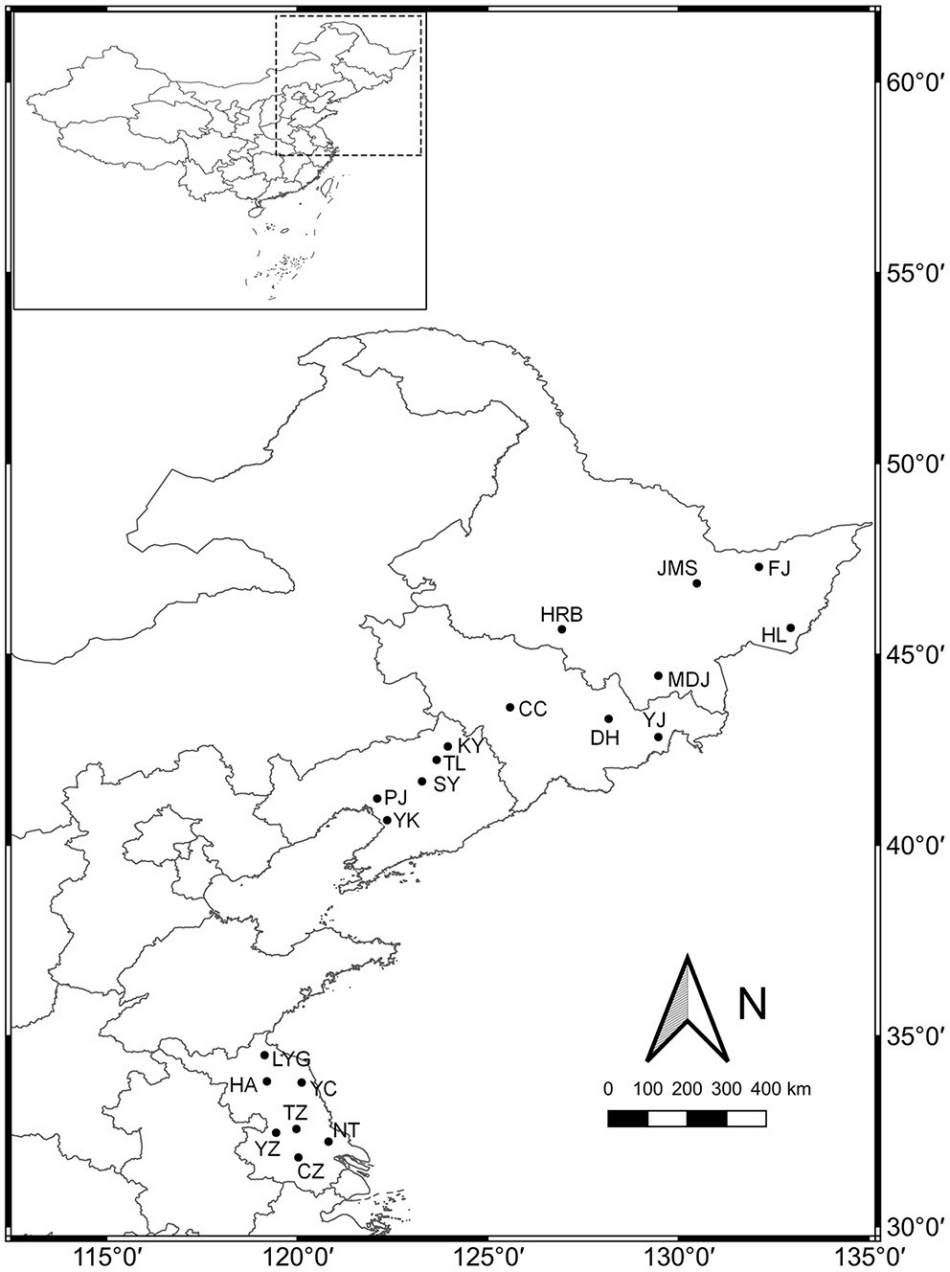
Sampling sites for 20 weedy rice populations investigated in northeastern and eastern China.

A total of 20 weedy rice populations located in Heilongjiang (HLJ), Jilin (JL), Liaoning (LN), and Jiangsu (JS) provinces, respectively, were investigated (Figure 1). The seeds of 30 weedy rice individuals and 3–5 co-existing cultivated rice individuals were collected from each population, and put in separate paper bags. The bags were stored in sorting boxes with silica gel at room temperature. Serial fixed-point investigation and sampling were conducted in 2013, 2015, and 2017, respectively. The seeds collected in 2013 and 2015 were used respectively in the common garden experiments.

### Seed Germination Experiment

To validate the divergence in germination behavior under different temperature regimes, the seeds of two weedy rice populations from each province were used for germination test (Supplementary Table 1). The seeds of each population were divided into nine groups, with 50 seeds in each group. Seeds of each group were sown in a petri dish with 10 mL pre-cooled distilled water and two filter papers. The dishes were then placed in dark growth chambers at 9, 12, and 15°C, respectively, with three dishes for each population under each condition. Seeds were considered germinated when radicle protrusion was visible. The number of germinated seeds were counted daily for a period of 30 days. Germination tests were performed three times at 6, 12, and 24 months after collection, respectively, to assess the potential effects of after-ripening period on seed germination. To evaluate the stability of the adaptive divergence in germination behavior, the seeds collected from common garden experiments were also subjected to germination tests by using the method mentioned above.

### Whole-Genome Resequencing (WGS) and SNP Calling

Based on phenotypic traits and germination characters of the weedy rice seeds collected, individuals from six populations (HLJ-2, JL-1, JL-2, LN-1, JS-1, JS-2) were used for whole genome resequencing (Supplementary Data 1). Twenty individuals were chosen randomly from each of the populations. Genomic DNAs were isolated from the fresh leaf tissue of seedlings using DNAsecure Plant Kit (TIANGEN, Beijing, China). DNA quality was checked by 1.2% agarose gel and NanoDrop 2000c spectrophotometer (Thermo Scientific). Paired-end libraries were constructed and sequenced on an Illumina HiSeq2000 system according to the manufacturer's instructions by Novogene (Beijing, China).

Reads were filtered and trimmed by Trimmomatic v0.36 (Bolger et al., 2014) with the recommended parameters (ILLUMINACLIP:TruSeq3-PE.fa:2:30:10 LEADING:3 TRAILING:3 SLIDINGWINDOW:4:15 MINLEN:36) in the software manual, and mapped to the *O. sativa* ssp *japonica* reference genome (Nipponbare, IRGSP-1.0, http://rapdb.dna.affrc.go.jp/) (Kawahara et al., 2013) using BWA v0.7.10 (Li and Durbin, 2009) with default settings. The resulting SAM files were converted to BAM files by SAMtools v1.7 (Li et al., 2009). Picard v1.119 (https://broadinstitute.github.io/picard/) was used to sort BAM files and mark PCR duplicates. The reads were then realigned around InDels with GATK IndelRealigner (Mckenna et al., 2010). SNP calling was performed using bcftools v1.7 (Li, 2011) with a minimum mapping quality of 20 and a minimum base quality of 20. Raw variants were further filtered with DP ≥ 5 and QUAL ≥ 30 by a in-house perl script. All detected SNPs were annotated with SnpEff v4.11 (Cingolani et al., 2012) based on the IRGSP-1.0 annotation.

### Identification of SNPs Potentially Involved in Local Adaptation

Genetic differentiation between two populations was quantified as absolute allele frequency difference (AFD) (Berner, 2019) across genome-wide SNPs detected in weedy rice. The 32 million SNP dataset of 3,024 cultivated rice based on whole genome resequencing were downloaded from Rice SNP-Seek Database (http://snp-seek.irri.org/) (Alexandrov et al., 2015). The SNP subset dataset of 121 *japonica* and 330 *indica* rice varieties originated from China at shared SNP loci were extracted from the total dataset for comparison. To reduce the bias of allele frequency estimation due to missing data, only SNP sites having sequencing reads from at least five individuals in each population were retained for AFD calculation. AFDs between the weedy rice from northeastern China and japonica rice varieties was first calculated. Based on the distribution of AFD values, the SNPs from the top 5% (AFD = 0.490) of the genome-wide AFD distribution in each population comparison were considered as significantly divergent SNPs (Laurentino et al., 2020). Then, AFDs between weedy rice populations from northeastern China and eastern China were calculated. To ensure comparability, the same threshold value was used to filter out SNPs showing no significant divergence between the weedy rice from two different regions. Finally, the SNPs potentially underlying adaptation were predicted by identifying the allele with elevated frequency in the weedy rice populations from northeastern China compared to those from eastern China and *japonica* rice.

### Gene Expression Analysis

The seeds collected from the populations HLJ-2, LN-1, and JS-2 were germinated at 6 months after collection in a dark incubator at 12°C. Seeds were collected respectively at 4 days after imibibition, the day just before radicle emergence (about 9 days after imbibition), and the time of radicle protrusion. Because the seeds from JS-2 did not germinate at all until 30 days after sowing, we put the dish in a dark growth chamber at 28°C to collect the germinated seeds. Total RNAs were extracted using the method described by Li and Trick (2005) with some modifications. DNase treatment was performed with RNase-free DNase (TIANGEN, Beijing, China). Total RNAs were purified and concentrated with RNeasy MiniElute Cleanup Kit (QIAGEN, Hilden, Germany). RNA quality was checked by NanoDrop 2000c spectrophotometer and gel electrophoresis. RNA integrity was further verified by Agilent BioAnalyzer 2100 (Agilent Technologies). Nine libraries were constructed using Ion Total RNA-Seq kit v2 (Life Technologies) according to the manufacturer's instructions and sequenced on an Ion Proton platform (Life Technologies) in BGI (Shenzhen, China).

After removal of adapter sequences, reads with length <30 were discarded. Average quality of 20 bases from 3′ end was calculated until average quality is larger than 9, then the bases that have been counted were trimmed. The resulting clean reads were mapped against the *japonica* rice reference genome (Nipponbare, IRGSP-1.0) with the TMAP v3.4.1 software (Life Technologies) and two mismatches were allowed. Gene expression levels were calculated by the RPKM method. Differentially expressed genes (DEGs) between samples were identified by the DEGseq package (Wang et al., 2010) in R. The false discovery rate (FDR) control method (Benjamini and Yekutieli, 2001) was adopted to correct *p*-values in multiple hypothesis tests. A gene was considered to be differentially expressed between two samples if it had an absolute value of |log2Ratio| ≥ 1 and an FDR < 0.001. GO enrichment analyses for DEGs and summary of the enriched GO terms were conducted with the topGO package in R Bioconductor and the REVIGO web server (Supek et al., 2011), respectively.

### Validation of SNP Allele Frequency and the Expression Pattern of Candidate Genes

Seeds from populations HLJ-2, LN-1, JS-1, and JS-2 were used for targeted SNP genotyping to validate the SNP allele frequency determined by WGS. Two other populations, HLJ-1 and LN-2, which were not included in WGS were also selected for SNP genotyping for evaluating the spread of potential adaptive alleles in northeastern populations. Thirty weedy rice individuals and one co-existing cultivated rice individual were used for each population. Genomic DNAs were extracted using the same method as described above. Genotyping was performed by applying the Sequenom MassArray platform in BGI (Shenzhen, China). The information of the 10 potentially adaptive SNPs genotyped by MassArray was listed in Supplementary Table 2. The genotype data of 451 cultivated rice varieties from China (330 *indica* varieties and 121 *japonica* varieties) at these SNP loci were extracted from previously downloaded SNP dataset (http://snp-seek.irri.org/). The whole genome resequencing data of 44 Asian wild rice (*O. rufipogon*) individuals from different natural populations in China (Xia et al., 2019) were also used for tracing the origin of potential beneficial SNPs.

The expression dynamics of five genes possessing distinct region-specific dominant alleles were validated by qRT-PCR. Seeds were germinated in a dark chamber at 9°C and collected at 0.5, 1, 2, 4, 6, 8, 10, 12, 14, 16, 18, and 20 d, respectively, after sowing. Total RNAs were extracted using the same method as described above. First-stranded cDNA was synthesized with PrimeScript RT Master Mix Perfect Real Time (TaKaRa, Dalian, China). Gene specific primer sequences used for qRT-PCR were listed in Supplementary Table 3. RT-PCR was performed on the Roche LightCycler96 system using SYBR® Premix Ex TaqTM II (TliRNaseH Plus) (TaKaRa, Dalian, China). UBQ5 was selected as the reference gene based on RNA-Seq profiles. Each qRT-PCR was performed on three biological replicates with three technical replicates each. Relative expression levels were calculated using the 2^−Δ*ΔCt*^ method. Seeds of the weedy rice from Jiangsu at 12 d after imbibition were used as controls to calibrate relative gene expression.

## Results

### Variations in Seed Germination Under Different Temperatures

**Figure 2:**
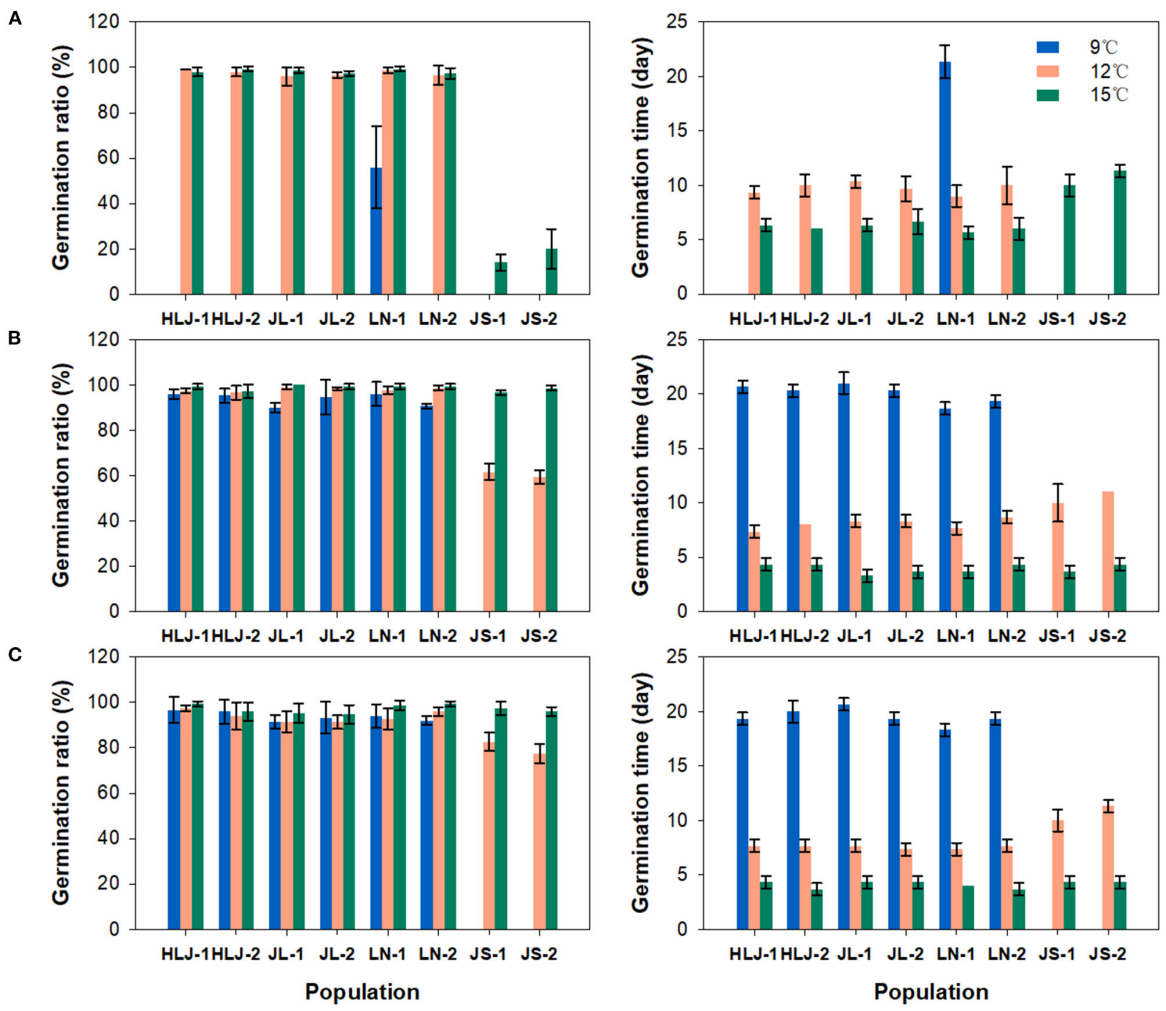
Germination ratio (left) and germination time (right) of weedy rice seeds from 8 populations at 9, 12, and 15°C, respectively. Vertical bars indicate the standard error of the mean. Germination tests were conducted at 6 **(A)**, 12 **(B)**, and 24 **(C)** months after collection, respectively.

Significant differences in seed germination were observed not only between the seeds of different populations from different regions, but also between the seeds from the same population with different storage time. In the first seed germination experiment (Figure 2A), only a few of the seeds collected from LN-1 population germinated at 9°C, taking more than 20 days. The seeds from northeastern China showed nearly 100% germination at 12 and 15°C, while those from the eastern China region did not germinate at 12°C and showed a lower germination ratio ranging from 10.0 to 30.0% at 15°C. Compared to the results of the first seed germination experiment, all populations from northeastern China showed very high germination (>90%) at 9°C in the second germination experiment (Figure 2B). The seeds from eastern China populations still showed no germination at 9°C but showed moderate germination ratio range from 56.0 to 64.0% at 12°C and exhibited almost complete germination at 15°C during the second germination experiment. Similar patterns of germination were found in the third seed germination experiment (Figure 2C). In accompany with variations in germination ratio, changes in germination time were also observed between populations at different temperatures. In general, seeds germinated more slowly under lower temperature, and the seeds from northeastern populations germinated faster than those from eastern populations. After 6 months storage, the germination time tends to be decreased in all populations under different temperatures in the second germination experiment, possibly suggesting a small degree of after-ripening in the weedy rice seeds though they can germinate immediately after harvest under favorable conditions. There is no significant difference between the results of the second and the third germination experiments. The stability of divergence in germination behavior between northeastern and eastern weedy rice populations was verified by the results of germination tests of the seeds collected from common garden experiments (Supplementary Figure 1).

### Region-Specific SNPs Potentially Underlying Adaptation

A total of 4,489,300 high-quality SNPs were identified in all weedy rice samples by WGS. Among them, 4,082,695 SNPs were retained after filtering and used for calculating the AFD value among different populations. A relatively lower level of differentiation was found between the northeastern weedy rice populations and *japonica* rice varieties (mean AFD = 0.0701). However, numerous SNP sites stood out from this background level of differentiation, with separate alleles reaching fixation in weedy rice populations and *japonica* rice varieties, respectively. The SNPs from the top 5% of the AFD distribution (AFD > 0.490) were considered as outliers and potential targets of adaptation (Supplementary Figure 2A). Greater differentiation was found between the weedy rice populations from northeastern and those from eastern regions, with a mean AFD value of 0.462 across all genome SNP sites (Supplementary Figure 2B). The same AFD threshold of 0.490 was used to identify high-differentiation SNPs between the weedy rice populations from northeastern and eastern China. After checking the direction of allele frequency changes, a total of 66,327 SNPs were found to show significantly higher allele frequencies in the weedy rice populations from northeastern China than in those from eastern China and in *japonica* rice varieties. Of them, 4,680 were annotated as non-synonymous variants or located in the UTR regions of 2,173 genes.

### Region-Specific DEGs During Germination

A total of 235,937,605 single-end clean reads of nine libraries were obtained. Around 21.2 million reads per library were uniquely mapped to the Nipponbare reference genome (IRGSP-1.0). In total, expressions of 29,204 genes were detected in at least one sample. The average number of expressed genes was 20,682 per sample (Supplementary Table 4).

Comparing with the JS samples, genes that were commonly up- or down-regulated in both HLJ and LN samples at the same germination stage were identified as region-specific DEGs. A total of 6,459, 4,697, and 7,512 DEGs were identified at the three germination stages, respectively. Most genes were merely up- or down-regulated at a single stage (Supplementary Figure 3). There were 35 and 44 genes that were constantly up- or down-regulated, respectively, in northeastern weedy rice across all three germination stages. GO enrichment analyses for DEGs revealed functional distinctions between the genes differentially expressed at various stages of germination (*p*-value < 0.05, Supplementary Data 2, Supplementary Figures 4–9).

### DEGs Harboring Potential Beneficial SNPs and Genotype Frequencies

**Figure 3:**
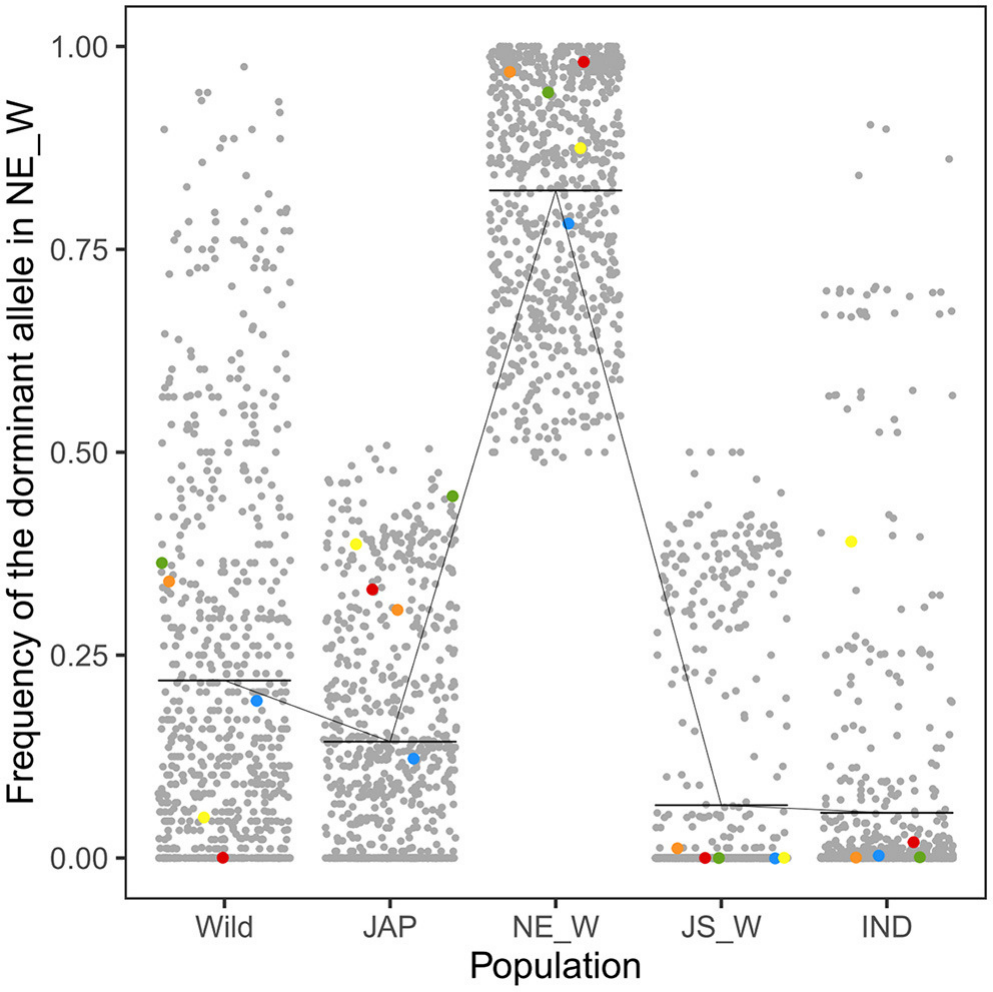
Frequencies of the northeastern weedy rice-specific predominant alleles in wild rice (Wild), *japonica* rice (JAP), northeastern weedy rice (NE_W), Jiangsu weedy rice (JS_W), and *indica* rice (IND) populations. Each dot represents a SNP allele. The horizontal bars indicate the mean allele frequency in each population. The red, blue, yellow, green, and orange dots represent the alleles located in *Os03g0122600, Os10g0136150, Os10g0155800, Os11g0191300*, and *Os08g0227200*, respectively. Their espression patterns were validated by qRT-PCR.

**Figure 4:**
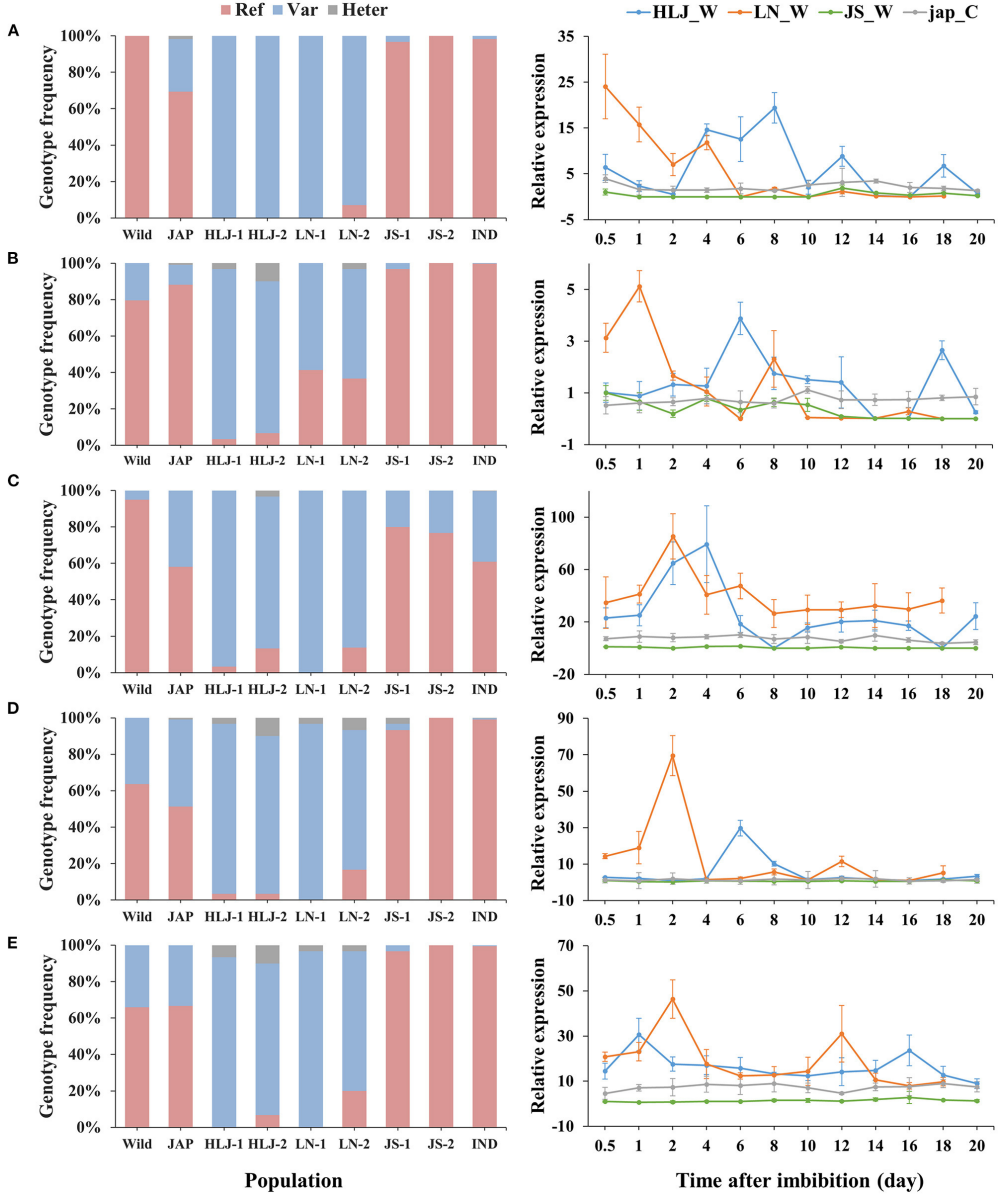
Genotype frequencies (left) and expression patterns (right) of 5 genes containing potential adaptive SNPs. **(A–E)** represent *Os03g0122600, Os10g0136150, Os10g0155800, Os11g0191300, Os08g0227200*, respectively. Ref, the homozygote of the reference base; Var, the homozygote of the variant base; Heter, the heterozygote of the reference and variant bases. HLJ_W, weedy rice from HLJ province; LN_W, weedy rice from LN province; JS_W, weedy rice from JS province; jap_C, the co-existing japonica rice.

Integrated analysis of the WGS and RNA-Seq data revealed 314 genes that were up-regulated in the northeastern weedy rice populations before seed germination, with each gene containing at least one highly differentiated SNP in the exon region. A total of 768 SNPs were located in the 314 up-regulated genes. The predominant alleles at these SNP loci in the northeastern weedy rice populations were distinct from those of the eastern populations and *japonica* rice varieties, but most of them were presented in cultivated *japonica* rice with medium and low frequencies (Figures 3, 4-left). Some of the northeastern weedy rice predominant alleles could even be traced back to wild rice with different frequencies, suggesting that the potential adaptive alleles may already exist in wild rice or were produced during cultivated rice domestication. A few of the SNP alleles predominant in the northeastern weedy rice were also present in the eastern weedy rice populations and in *indica* rice, mostly at lower frequencies. The allele frequency and genotype distribution of the SNPs contained in 10 most significantly differentially expressed genes were validated by targeted SNP genotyping. The results showed no significant differences in the allele frequencies estimated by WGS (paired *t*-test, *p*-value > 0.05). Moreover, the results demonstrated that the northeastern predominant alleles identified by WGS were also predominant in the northeastern weedy rice populations that were not subjected to WGS (Figure 4-left).

### Dynamic Expression of Candidate Adaptive Genes

The expression patterns of 10 genes subjected to targeted SNP genotyping assay were validated by qRT-PCR. Five of them showed consistent up-regulation at the early stage of germination in the samples from the northeastern region, that was clearly distinct from those of the eastern weedy rice and co-existing cultivars (Figure 4-right). The expression peaks of the weedy rice from LN occurred a little earlier than those of HLJ weedy rice, that was consistent with the slight differences in germination time between LN and HLJ weedy rice at 9°C. At later germination stages, the expression levels of all genes fluctuated and maintained at lower levels either a bit higher or temporally lower than those of JS weedy rice and the co-occurring cultivated rice. Of these genes, *Os03g0122600* encodes a putative MADS-box-containing transcription factor, homologous to *Arabidopsis SOC1*. *Os10g0155800, Os11g0191300*, and *Os08g0227200* encode a leucine-rich repeat receptor-like protein kinase, a DNA topoisomerase 2-binding protein and a BTB/POZ and MATH domain-containing protein, respectively, while *Os10g0136150* encodes an uncharacterized protein (Figures 4A–E). The SNP of *Os03g0122600* was located in the 3′-untranslated region. Instead, the other genes each contained a non-synonymous SNP in coding regions.

## Discussion

Weedy rice derived from cultivated progenitors developed an array of weed-adaptive traits to enhance their survival and persistence in the semi-natural habitats in agricultural landscapes. Convergent evolution has been described for some weedy traits, such as seed shattering and pericarp pigmentation, in populations with separate origins. However, unlike the weedy rice originated through exoferality in other regions of China, the weedy rice plants distributed in eastern and northeastern China did not diverge from their cultivated ancestors by reverting to the pre-domestication trait of seed dormancy during feralization. Instead, they developed a strategy to keep seeds from random germination under unfavorable conditions by sensing the appropriate ambient temperature cues. Subsequent population divergences have also been detected in the critical temperature for germination that were correlated with local habitat temperature at latitudinal gradient. The weedy rice seeds from higher latitudes could germinate at a lower temperature, whereas the cultivated rice seeds from corresponding latitudes and the weedy rice from JS did not show such a pattern. The region-specific differentiation in critical temperature ensured the feral plants to germinate at right time at different regions, which not only allowed the coordination of seed germination and plant establishment with the environment but also helped weedy rice plants to escape weed management tactics and outcompete the co-existing cultivated rice.

Integrated analysis of SNP allele frequency and expression data revealed genes potentially underpinning local adaptation. These genes possessed region-specific “weedy” alleles with high frequencies in northeastern populations, and were significantly up-regulated prior to germination at low temperature. Among them, *OsMADS50* (*Os03g0122600*) encoded a MADS-box-containing transcription factor, homologous to *Arabidopsis SOC1*. Previous studies have shown its function as a member of the molecular regulatory network of flowering by photoperiod and temperature, regulating long day (LD)-dependent flowering in rice (Ryu et al., 2009; Song and Luan, 2012). This function was similar to that of *Arabidopsis SOC1*, which mediated the crosstalk between cold response and flowering (Seo et al., 2009). Shao et al. (2019) showed that *OsMADS50* was also involved in the regulation of crown root development. However, up to now little is known about its functions in seed germination. As key regulators of plant development, MADS-box transcription factors seemed to be involved in almost every development process of plants, including seed development and germination (Smaczniak et al., 2012). It has been revealed that *Arabidopsis AGL21* acted as environmental surveillance of seed germination by regulating *ABI5* expression (Yu et al., 2017), while *ANR1* acted as a negative modulator of seed germination by activating *ABI3* expression (Lin et al., 2020). The *FLOWERING LOCUS C* (*FLC*, also known as *AGL25*), a key regulator of flowering, has also been reported to be involved in temperature-dependent seed germination by influencing the ABA catabolic and GA biosynthetic pathways (Chiang et al., 2009). The results of this study suggested that *OsMADS50* might play a vital role in integrating environmental information to regulate seed germination, and underpin the rapid local adaptation of weedy rice in germination behavior, although the mechanisms of function and regulation remained to be characterized. The potential role of *OsMADS50* as a thermo sensor was supported by other studies. Papaefthimiou et al. (2012) showed that the *SOC1*-like homolog in barley, *HvSOC1-like1* was induced in two winter barley cultivars after vernalization treatment, and they predicted that the presence of the *HvSOC1-like1* transcripts at the later stages of seed development might imply a role in the processes of dormancy breaking and germination. Voogd et al. (2015) also showed the role of kiwifruit *SOC1*-like genes in dormancy break, while Trainin et al. (2013) demonstrated that *ParSOC1* was linked with chilling requirements for dormancy break in apricot. Given that the expression of *ParSOC1* was sensitive to the environment with respect to the daily cycle in apricot, Trainin et al. (2013) also suggested that *ParSOC1* might be part of a pathway that modulates circadian rhythms in response to environmental cues and function as a circadian clock gene to be involved in dormancy break.

The function of other genes harboring potentially adaptive “weedy” alleles have not yet been well-characterized, but all the four genes subjected to genotyping and validation by qRT-PCR were predicted to be involved in response to abiotic stress based on GO annotations. *Os08g0227200* encoded an MATH-BTB domain containing protein which could act as a substrate-specific adaptor to bind with the E3 ubiquitin ligase component Cullin-3 to target protein for ubiquitination (Bauer et al., 2019). Juranić and Dresselhaus (2014) reported an expanded and highly divergent group of MATH-BTB proteins in different grass species. Functional analyses of the core-clade plant MATH-BTB proteins revealed their interaction with transcription factors involved in plant stress tolerance (Weber and Hellmann, 2009; Lechner et al., 2011). *Os10g0155800* encoded a leucine-rich repeat receptor-like protein kinase, predicted to be involved in stress related signal transduction pathway (Hossain et al., 2016). *Os11g0191300* was a homolog of *Arabidopsis MEI1* which has been reported to participate in DNA repair events (Mathilde et al., 2003). *Os10g0136150* encoded an uncharacterized F-box domain-containing protein. Although the specific roles of these genes remained to be characterized by experiments, the extreme allele frequency differences they exhibited between eastern and northeastern populations, as well as the unusual correlation between allele frequencies, allele-specific gene expression and feral phenotypes, suggested a polygenic nature of adaptation in seed germination behavior, and that weedy rice underwent rapid evolutionary changes at these loci.

Trancing the origin of potential adaptive alleles based on genomic sequences revealed the presence of most selected alleles in both wild and cultivated rice genomes, with a few alleles (such as the “weedy” allele of *OsMADS50*) being present in cultivated but not in wild rice genomes, indicating that weedy rice drew upon the cryptic genetic variation accumulated within wild and cultivated rice genomes to respond to environmental and anthropogenic pressures. The results also suggested the crucial role of standing genetic variation in facilitating the rapid adaptation of weedy rice to feral environments. It has been widely recognized that standing genetic variation could lead to faster adaptation than new mutations when environment changes, because adaptive alleles present as standing genetic variations were immediately available at higher frequencies, which might result in shorter fixation time (Barrett and Schluter, 2008). The potential adaptive alleles identified in this study were silent or cryptic in wild and cultivated rice genomes, showing no obvious phenotypic effects and retained with comparatively low frequencies, for that wild rice modulated seed germination by dormancy and cultivated rice seeds were usually germinated under human-controlled conditions. These hidden alleles were released in weedy rice in response to the feral selection regimes, promoting rapid evolution and population divergence in germination behavior. These alleles could thus be considered to be “conditionally neutral” that they were favored in one environment but neutral in others (Anderson et al., 2013). The conditional neutrality of the potential adaptive alleles might be reflected not only in their environment-dependent nature of activation but also in that their overt phenotypic expression was dependent upon the developmental context (Mee and Yeaman, 2019). Given that multiple potentially adaptive genes were detected with extreme levels of allele frequency divergence among populations, and that the predominate genotype combinations founded in northeastern weedy rice populations were absent from JS weedy rice and cultivated rice, we postulated that the rapid evolution of the novel germination strategy in weedy rice was probably reached via an increased covariance of a complementary set of loci with different fitness effects of alleles rather than via the allele frequency shift at a single locus (Le Corre and Kremer, 2012).

Crop domestication is a complex evolutionary process driven by strong artificial selection. Most of the domesticated traits are deleterious in natural environments but meet the demands of human. During domestication, plants mostly experienced severe genetic bottlenecks, leading to dramatic reduction in genetic diversity. Thus, it was usually recognized that domesticated organisms were incapable of rapid evolution, due to their genetic homogeneity and poor adaptive potential in environments outside of domesticated settings. The feralization process of crop species seems changing our understanding of the capacity of domesticated plants to evolve in the face of changing environments. Despite undergoing two successive bottleneck events associated respectively with domestication and de-domestication, weedy rice has rapidly evolved out of a novel strategy to precisely regulate seed germination via thermal sensing in response to regional differences in environmental temperature. This result indicated that feralization was not just a process of atavism and loss of domestication-related traits, but included the appearance of new traits that might facilitate ferality. Other studies have also shown that the reacquisition of wild-like traits during feralization was mostly not through changes at the loci related to domestication and that crop ferality was underpinned by novel and independent genetic mechanisms (Thurber et al., 2010, 2013; Qi et al., 2015). Together these findings support the view that domestication is not a dead end (Gering et al., 2019). The altered selection regimes imposed on feral populations can release the cryptic beneficial alleles preexisted in cultivated progenitor populations, leading to shifts in allele frequency from values observed as standing neutral variation in the ancestral population to the very high frequencies of predominant alleles in feral populations, and generate previously unobserved phenotypes. Genetic drift seemed to have a limited effect on the dynamics of allelic frequency in this study, since that the potential weed-adaptive alleles approached fixation in northeastern weedy rice populations but not in eastern populations. Weedy rice distributed in different areas were subjected to selection pressures not only from local environmental stressors (such as regional ambient temperatures), but also from the varied influence of regional agricultural practices for rice planting, for them coexisting in close proximity with cultivated rice in crop fields instead of returning to truly wild habitats. Dual selection pressures placed on northeastern and eastern weedy rice populations have driven rapid evolution and population divergence in germination behavior.

## Conclusion

Feral species have attracted the attention of researchers since Darwin (Mabry et al., 2021). Rapid accumulation of complete genome sequences in the last decades have aroused great concern about the evolutionary processes and mechanisms underlying feralization (Wu et al., 2021). Routes leading to the transition of a population from domestic to feral are diverse (Qiu et al., 2020). While many studies have shown phenotypic changes in feral plants similar to their wild ancestors, it should be noted that the evolution of ferality is not necessarily based on returning to ancestral states. The altered selection regimes associated with the transition from cultivation to wild existence can expose the cryptic phenotypes by activating formerly silent alleles, facilitating swift responses to the feral selection regimes and promoting the evolution and persistence of weedy forms.

## Data Availability Statement

The original contributions presented in the study are publicly available. This data can be found here: NCBI Sequence Read Archive (SRA) (http://www.ncbi.nlm.nih.gov/sra) under the BioProject ID PRJNA723638.

## Author Contributions

JY, BL, and LL designed the research. CZ, YF, MW, JJ, and GL sampled materials. CZ performed laboratory work, analyzed sequence data, and drafted the manuscript. YW, WZ, and ZS advised on design and discussed the results. All co-authors read and edited the manuscript. All authors contributed to the article and approved the submitted version.

## Conflict of Interest

The authors declare that the research was conducted in the absence of any commercial or financial relationships that could be construed as a potential conflict of interest.

## References

[B1] Alexandrov N. Tai S. S. Wang W. S. Mansueto L. Palis K. Fuentes R. R.. (2015). SNP-Seek database of SNPs derived from 3000 rice genomes. Nucleic Acids Res. 43, D1023–D1027. 10.1093/nar/gku1039 25429973 PMC4383887

[B2] Allaby R. G. Stevens C. Lucas L. Maeda O. Fuller D. Q. (2017). Geographic mosaics and changing rates of cereal domestication. Philos. Trans. R. Soc. Lond. B Biol. Sci. 372:20160429. 10.1098/rstb.2016.0429 29061901 PMC5665816

[B3] Anderson J. T. Lee C. R. Rushworth C. A. Colautti R. I. Mitchell-Olds T. (2013). Genetic trade-offs and conditional neutrality contribute to local adaptation. Mol. Ecol. 22, 699–708. 10.1111/j.1365-294X.2012.05522.x 22420446 PMC3492549

[B4] Barrett R. D. H. Schluter D. (2008). Adaptation from standing genetic variation. Trends Ecol. Evol. 23, 38–44. 10.1016/j.tree.2007.09.008 18006185

[B5] Bauer N. Skiljaica A. Malenica N. Razdorov G. Klasic M. Juranic M.. (2019). The MATH-BTB protein *TaMAB2* accumulates in ubiquitin-containing foci and interacts with the translation initiation machinery in *Arabidopsis*. Front. Plant Sci. 10:1469. 10.3389/fpls.2019.01469 31824527 PMC6883508

[B6] Benjamini Y. Yekutieli D. (2001). The control of the false discovery rate in multiple testing under dependency. Ann. Stat. 29, 1165–1188. 10.1214/aos/1013699998

[B7] Berner D. (2019). Allele frequency difference AFD-an intuitive alternative to *F*_ST_ for quantifying genetic population differentiation. Genes 10:308. 10.3390/genes10040308 31003563 PMC6523497

[B8] Bolger A. M. Lohse M. Usadel B. (2014). Trimmomatic: a flexible trimmer for Illumina sequence data. Bioinformatics 30, 2114–2120. 10.1093/bioinformatics/btu170 24695404 PMC4103590

[B9] Burke J. M. Burger J. C. Chapman M. A. (2007). Crop evolution: from genetics to genomics. Curr. Opin. Genet. Dev. 17, 525–532. 10.1016/j.gde.2007.09.003 17933510

[B10] Cao Q. J. Lu B. R. Xia H. Rong J. Sala F. Spada A.. (2006). Genetic diversity and origin of weedy rice (*Oryza sativa* f. *spontanea*) populations found in North-eastern China revealed by simple sequence repeat (SSR) markers. Ann. Bot. 98, 1241–1252. 10.1093/aob/mcl210 17056615 PMC3292271

[B11] Chiang G. C. K. Barua D. Kramer E. M. Amasino R. M. Donohue K. (2009). Major flowering time gene, *FLOWERING LOCUS C*, regulates seed germination in *Arabidopsis thaliana*. Proc. Natl. Acad. Sci. U. S. A. 106, 11661–11666. 10.1073/pnas.0901367106 19564609 PMC2710639

[B12] Cingolani P. Platts A. Wang L. L. Coon M. Nguyen T. Wang L.. (2012). A program for annotating and predicting the effects of single nucleotide polymorphisms, SnpEff: SNPs in the genome of *Drosophila melanogaster* strain w(1118); iso-2; iso-3. Fly 6, 80–92. 10.4161/fly.19695 22728672 PMC3679285

[B13] Delouche J. C. Burgos N. R. Labrada R. Gealy D. R. (2007). Weedy Rices: Origin, Biology, Ecology and Control. Rome: Food and Agriculture Organization of the United Nations.

[B14] Doebley J. F. Gaut B. S. Smith B. D. (2006). The molecular genetics of crop domestication. Cell 127, 1309–1321. 10.1016/j.cell.2006.12.006 17190597

[B15] Ellstrand N. C. Heredia S. M. Leak-Garcia J. A. Heraty J. M. Burger J. C. Yao L.. (2010). Crops gone wild: evolution of weeds and invasives from domesticated ancestors. Evol. Appl. 3, 494–504. 10.1111/j.1752-4571.2010.00140.x 25567942 PMC3352506

[B16] Eyre-Walker A. Gaut R. L. Hilton H. Feldman D. L. Gaut B. S. (1998). Investigation of the bottleneck leading to the domestication of maize. Proc. Natl. Acad. Sci. U. S. A. 95, 4441–4446. 10.1073/pnas.95.8.4441 9539756 PMC22508

[B17] Gering E. Incorvaia D. Henriksen R. Conner J. Getty T. Wright D. (2019). Getting back to nature: feralization in animals and plants. Trends Ecol. Evol. 34, 1137–1151. 10.1016/j.tree.2019.07.018 31488326 PMC7479514

[B18] Gressel J. (2005). Crop Ferality and Volunteerism. Boca Raton: CRC Press. 10.1201/9781420037999

[B19] Hossain M. R. Bassel G. W. Pritchard J. Sharma G. P. Ford-Lloyd B. V. (2016). Trait specific expression profiling of salt stress responsive genes in diverse rice genotypes as determined by modified significance analysis of microarrays. Front. Plant Sci. 7:567. 10.3389/fpls.2016.00567 27200040 PMC4853522

[B20] Hoyos V. Plaza G. Li X. Caicedo A. L. (2020). Something old, something new: evolution of Colombian weedy rice (*Oryza* spp.) through de novo de-domestication, exotic gene flow, and hybridization. Evol. Appl. 13, 1968–1983. 10.1111/eva.12955 32908598 PMC7463356

[B21] Huang Z. Y. Young N. D. Reagon M. Hyma K. E. Olsen K. M. Jia Y. L.. (2017). All roads lead to weediness: patterns of genomic divergence reveal extensive recurrent weedy rice origins from South Asian *Oryza*. Mol. Ecol. 26, 3151–3167. 10.1111/mec.14120 28345200

[B22] Juranić M. Dresselhaus T. (2014). Phylogenetic analysis of the expansion of the MATH-BTB gene family in the grasses. Plant Signal. Behav. 9:e28242. 10.4161/psb.28242 24614623 PMC4091423

[B23] Kawahara Y. De La Bastide M. Hamilton J. P. Kanamori H. Mccombie W. R. Ouyang S.. (2013). Improvement of the *Oryza sativa* Nipponbare reference genome using next generation sequence and optical map data. Rice 6:4. 10.1186/1939-8433-6-4 24280374 PMC5395016

[B24] Laurentino T. G. Moser D. Roesti M. Ammann M. Frey A. Ronco F.. (2020). Genomic release-recapture experiment in the wild reveals within-generation polygenic selection in stickleback fish. Nat. Commun. 11:1928. 10.1038/s41467-020-15657-3 32317640 PMC7174299

[B25] Le Corre V. Kremer A. (2012). The genetic differentiation at quantitative trait loci under local adaptation. Mol. Ecol. 21, 1548–1566. 10.1111/j.1365-294X.2012.05479.x 22332667

[B26] Lechner E. Leonhardt N. Eisler H. Parmentier Y. Alioua M. Jacquet H.. (2011). MATH/BTB CRL3 receptors target the homeodomain-leucine zipper *ATHB6* to modulate abscisic acid signaling. Dev. Cell 21, 1116–1128. 10.1016/j.devcel.2011.10.018 22172674

[B27] Li H. (2011). A statistical framework for SNP calling, mutation discovery, association mapping and population genetical parameter estimation from sequencing data. Bioinformatics 27, 2987–2993. 10.1093/bioinformatics/btr509 21903627 PMC3198575

[B28] Li H. Durbin R. (2009). Fast and accurate short read alignment with Burrows-Wheeler transform. Bioinformatics 25, 1754–1760. 10.1093/bioinformatics/btp324 19451168 PMC2705234

[B29] Li H. Handsaker B. Wysoker A. Fennell T. Ruan J. Homer N.. (2009). The sequence alignment/map format and SAMtools. Bioinformatics 25, 2078–2079. 10.1093/bioinformatics/btp352 19505943 PMC2723002

[B30] Li L. F. Li Y. L. Jia Y. L. Caicedo A. L. Olsen K. M. (2017). Signatures of adaptation in the weedy rice genome. Nat. Genet. 49, 811–814. 10.1038/ng.3825 28369039

[B31] Li Z. W. Trick H. N. (2005). Rapid method for high-quality RNA isolation from seed endosperm containing high levels of starch. BioTechniques 38, 872–876. 10.2144/05386BM05 16018547

[B32] Lin J. H. Yu L. H. Xiang C. B. (2020). ARABIDOPSIS NITRATE REGULATED 1 acts as a negative modulator of seed germination by activating *ABI3* expression. New Phytol. 225, 835–847. 10.1111/nph.16172 31491809

[B33] Mabry M. E. Rowan T. N. Pires J. C. Decker J. E. (2021). Feralization: confronting the complexity of domestication and evolution. Trends Genet. 4, 302–305. 10.1016/j.tig.2021.01.005 33546926

[B34] Mathilde G. Ghislaine G. Daniel V. Georges P. (2003). The *Arabidopsis MEI1* gene encodes a protein with five BRCT domains that is involved in meiosis-specific DNA repair events independent of *SPO11*-induced DSBs. Plant J. 35, 465–475. 10.1046/j.1365-313X.2003.01820.x 12904209

[B35] Mckenna A. Hanna M. Banks E. Sivachenko A. Cibulskis K. Kernytsky A.. (2010). The Genome Analysis Toolkit: a MapReduce framework for analyzing next-generation DNA sequencing data. Genome Res. 20, 1297–1303. 10.1101/gr.107524.110 20644199 PMC2928508

[B36] Mee J. A. Yeaman S. (2019). Unpacking conditional neutrality: genomic signatures of selection on conditionally beneficial and conditionally deleterious mutations. Am. Nat. 194, 529–540. 10.1086/702314 31490722

[B37] Papaefthimiou D. Kapazoglou A. Tsaftaris A. S. (2012). Cloning and characterization of *SOC1* homologs in barley (*Hordeum vulgare*) and their expression during seed development and in response to vernalization. Physiol. Plant. 146, 71–85. 10.1111/j.1399-3054.2012.01610.x 22409646

[B38] Qi X. S. Liu Y. Vigueira C. C. Young N. D. Caicedo A. L. Jia Y. L.. (2015). More than one way to evolve a weed: parallel evolution of US weedy rice through independent genetic mechanisms. Mol. Ecol. 24, 3329–3344. 10.1111/mec.13256 26031196

[B39] Qiu J. Jia L. Wu D. Y. Weng X. F. Chen L. J. Sun J.. (2020). Diverse genetic mechanisms underlie worldwide convergent rice feralization. Genome Biol. 21:70. 10.1186/s13059-020-01980-x 32213201 PMC7098168

[B40] Qiu J. Zhou Y. J. Mao L. F. Ye C. Y. Wang W. D. Zhang J. P.. (2017). Genomic variation associated with local adaptation of weedy rice during de-domestication. Nat. Commun. 8:15323. 10.1038/ncomms15323 28537247 PMC5477509

[B41] Reagon M. Thurber C. S. Gross B. L. Olsen K. M. Jia Y. L. Caicedo A. L. (2010). Genomic patterns of nucleotide diversity in divergent populations of US weedy rice. BMC Evol. Biol. 10:180. 10.1186/1471-2148-10-180 20550656 PMC2898691

[B42] Ryu C. H. Lee S. Cho L. H. Kim S. L. Lee Y. S. Choi S. C.. (2009). *OsMADS50* and *OsMADS56* function antagonistically in regulating long day (LD)-dependent flowering in rice. Plant Cell Environ. 32, 1412–1427. 10.1111/j.1365-3040.2009.02008.x 19558411

[B43] Seo E. Lee H. Jeon J. Park H. Kim J. Noh Y. S.. (2009). Crosstalk between cold response and flowering in *Arabidopsis* is mediated through the flowering-time gene *SOC1* and its upstream negative regulator *FLC*. Plant Cell 21, 3185–3197. 10.1105/tpc.108.063883 19825833 PMC2782271

[B44] Shao Y. L. Zhou H. Z. Wu Y. R. Zhang H. Lin J. Jiang X. Y.. (2019). *OsSPL3*, an SBP-domain protein, regulates crown root development in rice. Plant Cell 31, 1257–1275. 10.1105/tpc.19.00038 30940685 PMC6588302

[B45] Smaczniak C. Immink R. G. H. Angenent G. C. Kaufmann K. (2012). Developmental and evolutionary diversity of plant MADS-domain factors: insights from recent studies. Development 139, 3081–3098. 10.1242/dev.074674 22872082

[B46] Song Y. L. Luan W. J. (2012). Molecular regulatory network of flowering by photoperiod and temperature in rice. Rice Sci. 19, 169–176. 10.1016/S1672-6308(12)60037-724651369

[B47] Supek F. Bosnjak M. Skunca N. Smuc T. (2011). REVIGO summarizes and visualizes long lists of gene ontology terms. PLos ONE 6:e21800. 10.1371/journal.pone.0021800 21789182 PMC3138752

[B48] Thurber C. S. Jia M. H. Jia Y. L. Caicedo A. L. (2013). Similar traits, different genes? Examining convergent evolution in related weedy rice populations. Mol. Ecol. 22, 685–698. 10.1111/mec.12147 23205731

[B49] Thurber C. S. Reagon M. Gross B. L. Olsen K. M. Jia Y. L. Caicedo A. L. (2010). Molecular evolution of shattering loci in US weedy rice. Mol. Ecol. 19, 3271–3284. 10.1111/j.1365-294X.2010.04708.x 20584132 PMC2988683

[B50] Trainin T. Bar-Ya'akov I. Holland D. (2013). *ParSOC1*, a MADS-box gene closely related to *Arabidopsis AGL20*/*SOC1*, is expressed in apricot leaves in a diurnal manner and is linked with chilling requirements for dormancy break. Tree Genet. Genom. 9, 753–766. 10.1007/s11295-012-0590-8

[B51] Vigueira C. C. Olsen K. M. Caicedo A. L. (2013). The red queen in the corn: agricultural weeds as models of rapid adaptive evolution. Heredity 110, 303–311. 10.1038/hdy.2012.104 23188175 PMC3607111

[B52] Vigueira C. C. Qi X. S. Song B. K. Li L. F. Caicedo A. L. Jia Y. L.. (2019). Call of the wild rice: *Oryza rufipogon* shapes weedy rice evolution in Southeast Asia. Evol. Appl. 12, 93–104. 10.1111/eva.12581 30622638 PMC6304679

[B53] Voogd C. Wang T. C. Varkonyi-Gasic E. (2015). Functional and expression analyses of kiwifruit *SOC1*-like genes suggest that they may not have a role in the transition to flowering but may affect the duration of dormancy. J. Exp. Bot. 66, 4699–4710. 10.1093/jxb/erv234 25979999 PMC4507769

[B54] Wang L. K. Feng Z. X. Wang X. Wang X. W. Zhang X. G. (2010). DEGseq: an R package for identifying differentially expressed genes from RNA-seq data. Bioinformatics 26, 136–138. 10.1093/bioinformatics/btp612 19855105

[B55] Weber H. Hellmann H. (2009). *Arabidopsis thaliana* BTB/POZ-MATH proteins interact with members of the ERF/AP2 transcription factor family. FEBS J. 276, 6624–6635. 10.1111/j.1742-4658.2009.07373.x 19843165

[B56] Wu D. Lao S. Fan L. (2021). De-domestication: an extension of crop evolution. Trends Plant Sci. 26, 560–574. 10.1016/j.tplants.2021.02.003 33648850

[B57] Xia H. Luo Z. Xiong J. Ma X. S. Lou Q. J. Wei H. B.. (2019). Bi-directional selection in upland rice leads to its adaptive differentiation from lowland rice in drought resistance and productivity. Mol. Plant. 12, 170–184. 10.1016/j.molp.2018.12.011 30584948

[B58] Xia H. B. Xia H. Ellstrand N. C. Yang C. Lu B. R. (2011). Rapid evolutionary divergence and ecotypic diversification of germination behavior in weedy rice populations. New Phytol. 191, 1119–1127. 10.1111/j.1469-8137.2011.03766.x 21569036

[B59] Yu L. H. Wu J. Zhang Z. S. Miao Z. Q. Zhao P. X. Wang Z.. (2017). *Arabidopsis* MADS-box transcription factor *AGL21* acts as environmental surveillance of seed germination by regulating *ABI5* expression. Mol. Plant. 10, 834–845. 10.1016/j.molp.2017.04.004 28438576

[B60] Zhang S. Tian L. Li J. Wang C. Lee D. Peng R.. (2017). Morphological characterization of weedy rice populations from different regions of Asia. Mol. Plant Breed. 8, 52–64. 10.1007/s11032-017-0653-5

