# Supplementary material for: The New Is Old: Novel Germination Strategy Evolved From Standing Genetic Variation in Weedy Rice
Source: Front Plant Sci. 2021 Jun 21;12:699464. doi: 10.3389/fpls.2021.699464 (PMC8256273; doi:10.3389/fpls.2021.699464)
Supplement: Supplementary file 3 [file Data_Sheet_3.DOCX]

Supplementary Material

# Supplementary Figures and Tables

## Supplementary Figures


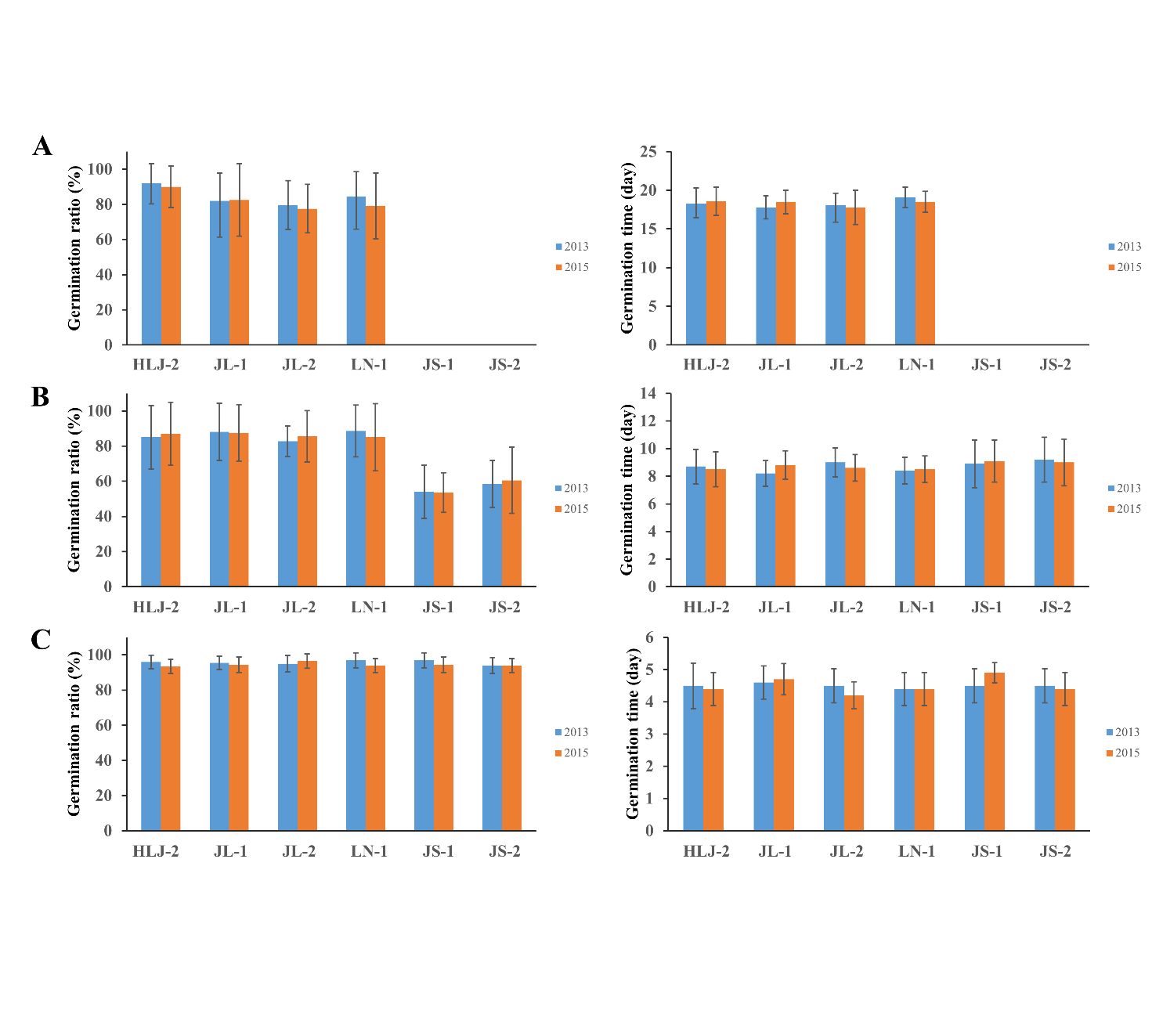


**Supplementary Figure 1.** Germination ratio (left) and germination time (right) of weedy rice seeds collected from common gardens. Three independent seed germination experiments conducted at 9℃ (A), 12℃ (B) and 15℃ (C), respectively.


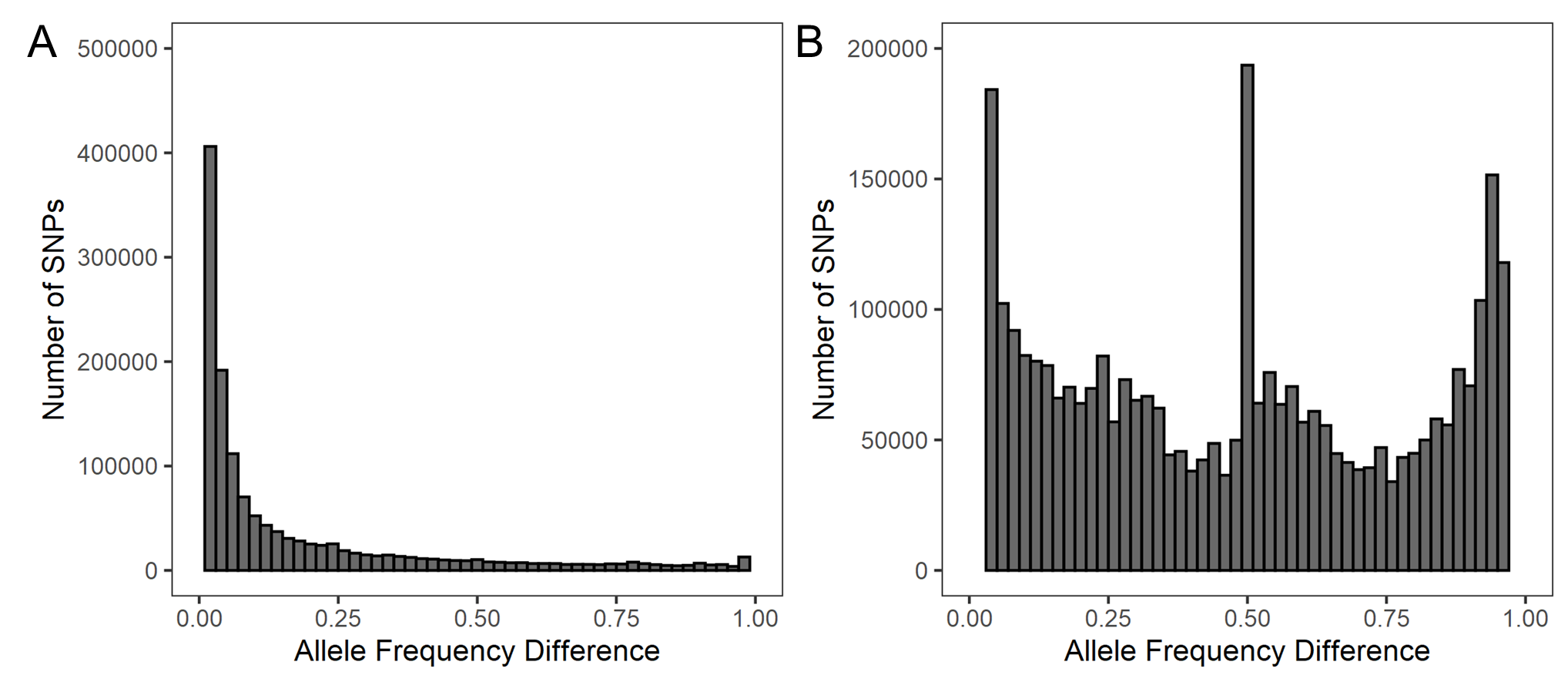


**Supplementary Figure 2.** Absolute allele frequency difference (AFD) distributions among different rice groups across 4.08 million SNP sites. **(A)** the AFD distribution between the northeastern weedy rice and its putative ancestor *japonica* rice varieties. **(B)** the AFD distribution between the northeastern weedy rice and the eastern weedy rice.


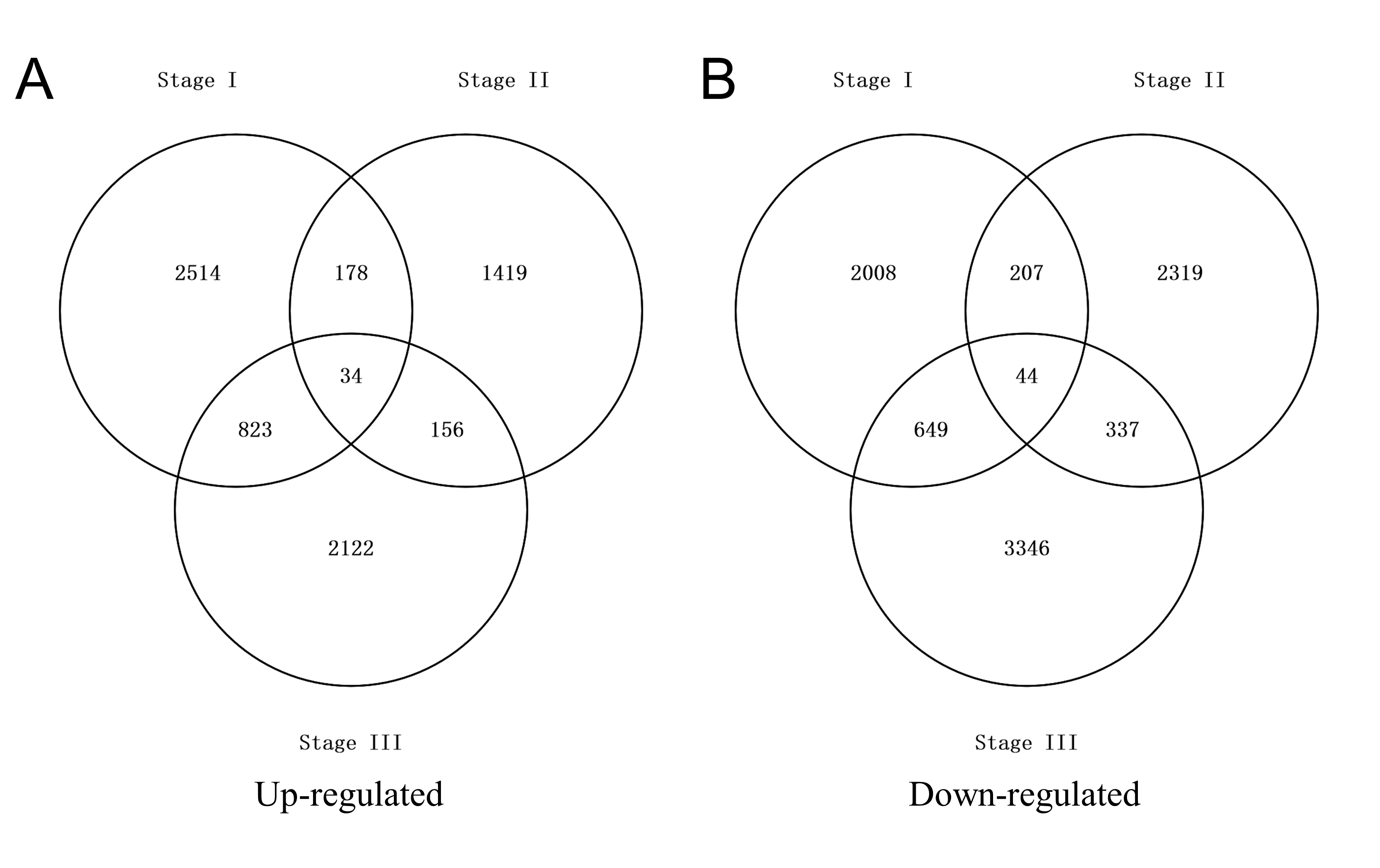


**Supplementary Figure 3.** Venn diagrams with the number of genes differentially expressed between northeastern and eastern weedy rice at three different germination stages. **(A)** Numbers of up-regulated gene in northeastern weedy rice samples compared to eastern weedy rice samples at three germination stages respectively. **(B)** Numbers of down-regulated gene in northeastern weedy rice samples compared to eastern weedy rice samples at three germination stages respectively.


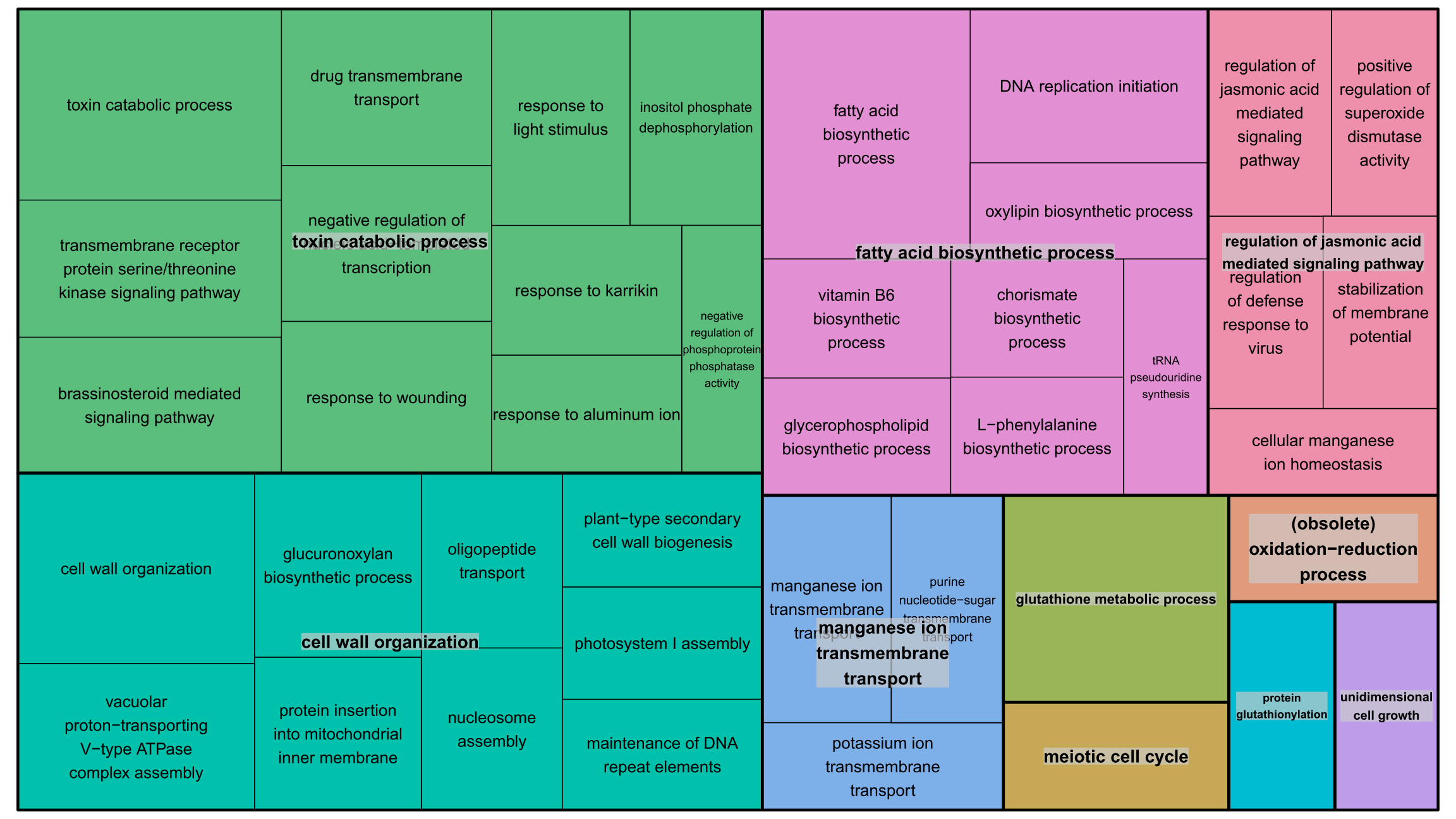


**Supplementary Figure 4.** Summarized results of enriched GO terms among the up-regulated genes in weedy rice from northeastern China at Stage I by REViGO.


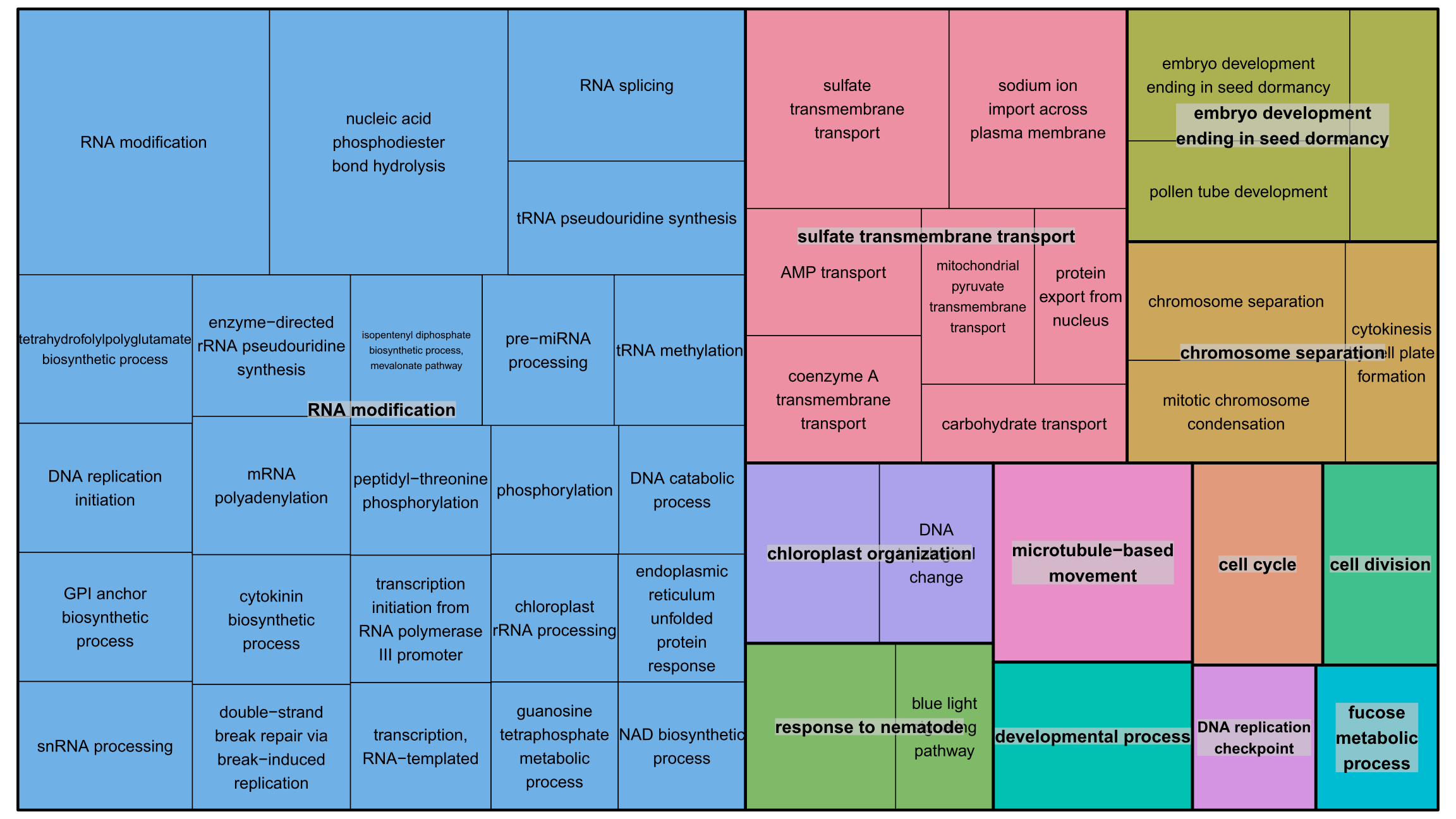


**Supplementary Figure 5.** Summarized results of enriched GO terms among the down-regulated genes in weedy rice from northeastern China at Stage I by REViGO.


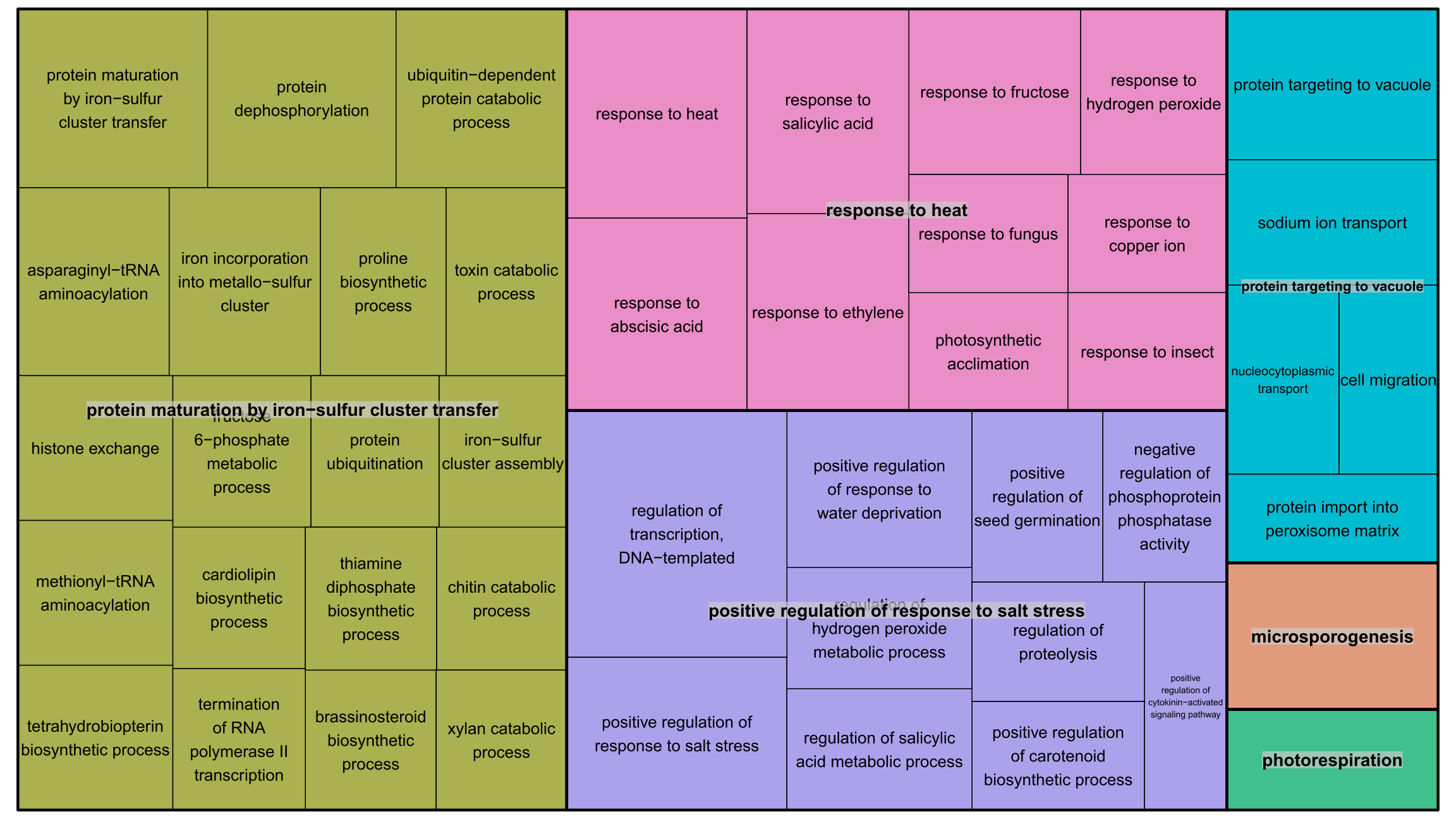


**Supplementary Figure 6.** Summarized results of enriched GO terms among the up-regulated genes in weedy rice from northeastern China at Stage II by REViGO.


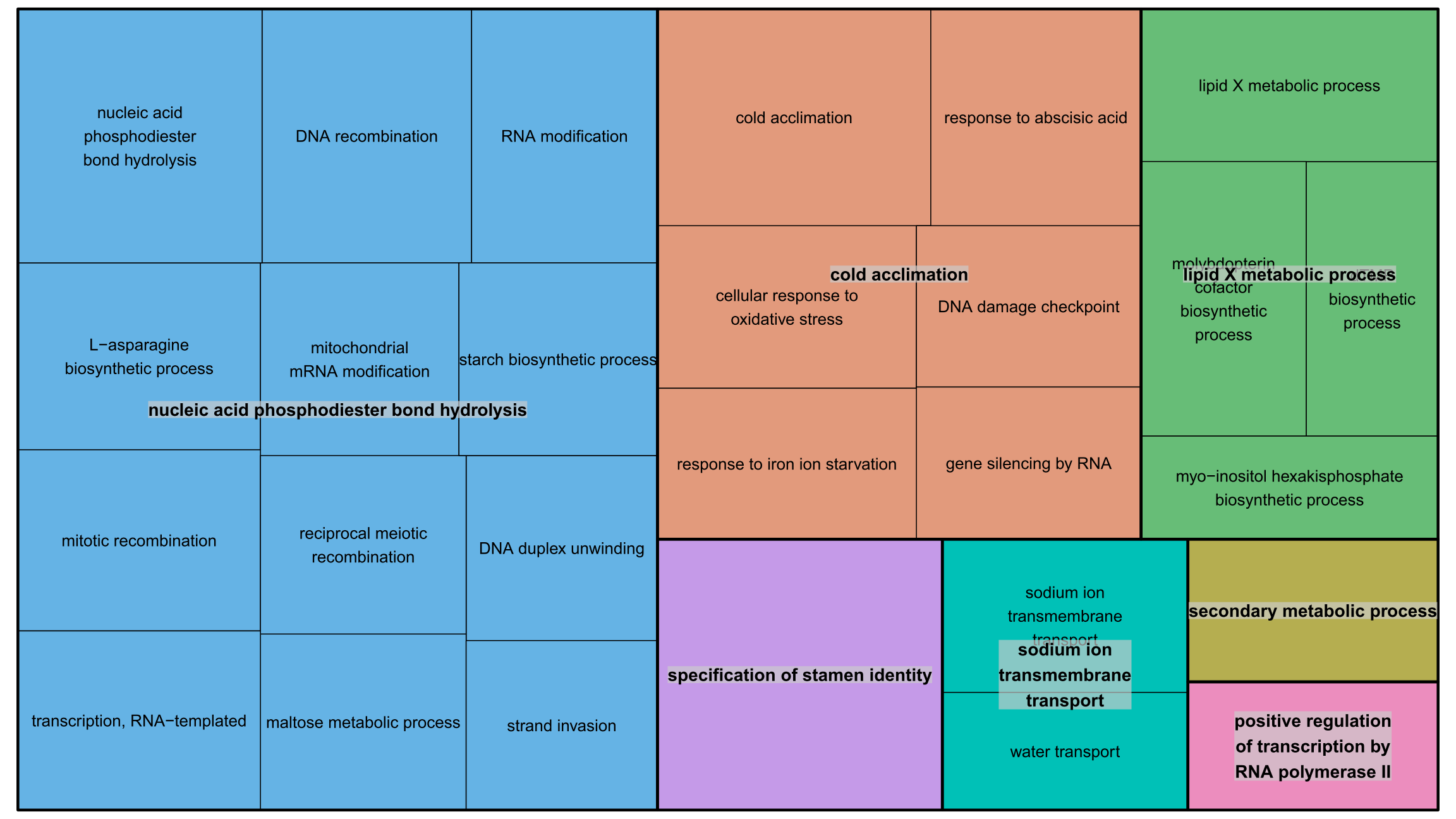


**Supplementary Figure 7.** Summarized results of enriched GO terms among the down-regulated genes in weedy rice from northeastern China at Stage II by REViGO.


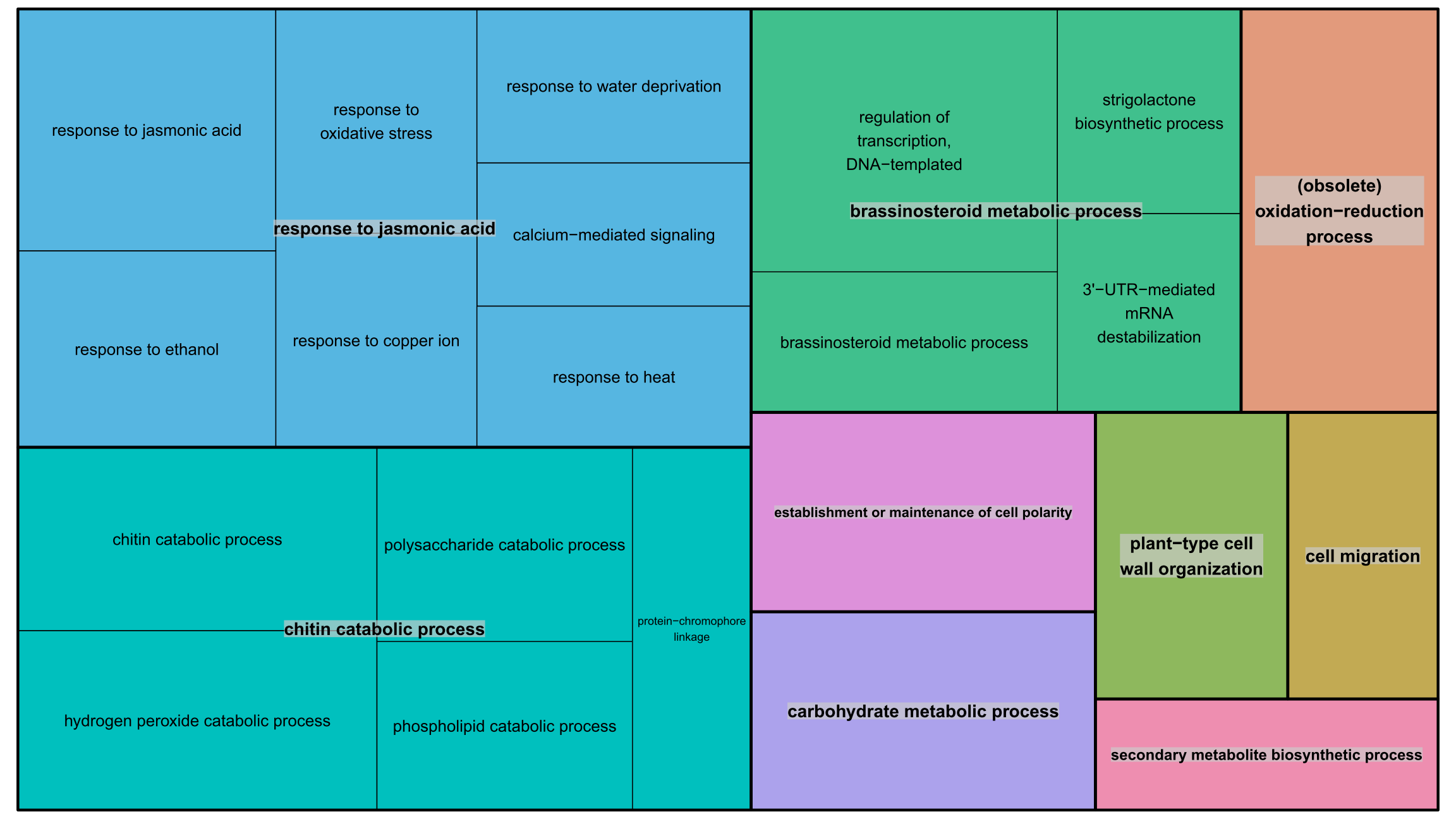


**Supplementary Figure 8.** Summarized results of enriched GO terms among the up-regulated genes in weedy rice from northeastern China at Stage III by REViGO.


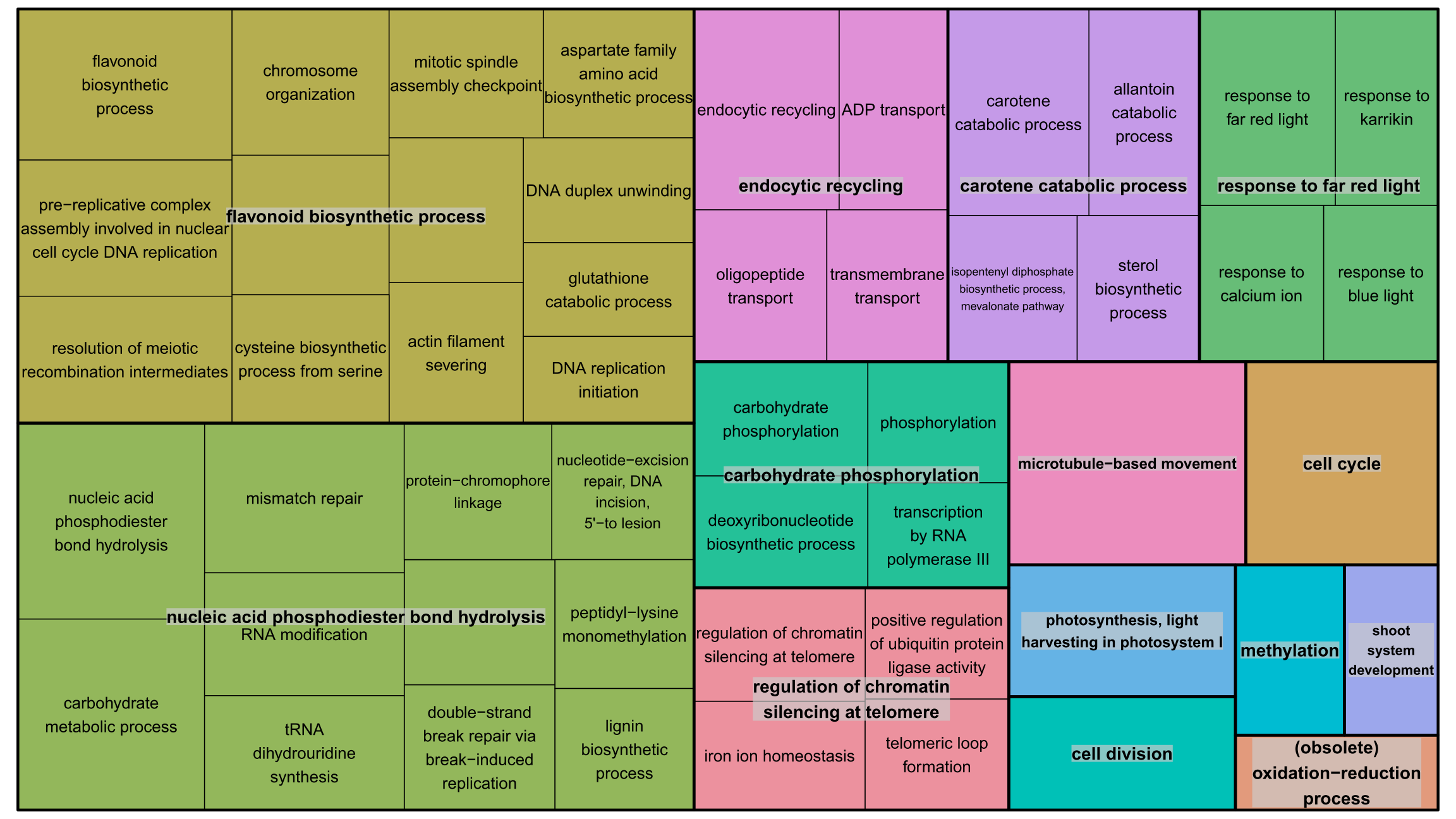


**Supplementary Figure 9.** Summarized results of enriched GO terms among the down-regulated genes in weedy rice from northeastern China at Stage III by REViGO.

## Supplementary Tables

**Supplementary Table 1**. Basic information of the weedy rice populations used in this study.

| Population | Region | Province | Location | Longitude (°) | Latitude (°) | Number of weedy rice individuals |
| --- | --- | --- | --- | --- | --- | --- |
| HLJ-1 | Northeastern China | Heilongjiang | Jiamusi | 130.4873 | 46.8570 | 30 |
| HLJ-2 | Northeastern China | Heilongjiang | Hulin | 132.9587 | 45.7448 | 30 |
| JL-1 | Northeastern China | Jilin | Dunhua | 128.1729 | 43.3057 | 30 |
| JL-2 | Northeastern China | Jilin | Yanji | 129.4774 | 42.8291 | 30 |
| LN-1 | Northeastern China | Liaoning | Kaiyuan | 123.9531 | 42.5822 | 30 |
| LN-2 | Northeastern China | Liaoning | Yingkou | 122.3619 | 40.6488 | 30 |
| JS-1 | Eastern China | Jiangsu | Yangzhou | 119.4474 | 32.4578 | 30 |
| JS-2 | Eastern China | Jiangsu | Changzhou | 120.0327 | 31.8103 | 30 |

**Supplementary Table 2.** Ten potentially adaptive SNP sites genotyped by targeted SNP genotyping.

| Chromosome | Position | REF | ALT | Gene | Region | AFD1 | AFD2 |
| --- | --- | --- | --- | --- | --- | --- | --- |
| chr01 | 304168 | A | T | Os01g0105700 | CDS | 0.650 | 0.981 |
| chr03 | 1270331 | C | G | Os03g0122600 | 3'UTR | 0.663 | 0.956 |
| chr03 | 13155925 | T | C | Os03g0351200 | 3'UTR | 0.659 | 0.781 |
| chr07 | 10175435 | G | T | Os07g0273900 | 3'UTR | 0.497 | 0.944 |
| chr08 | 7760912 | C | T | Os08g0227200 | CDS | 0.613 | 1.000 |
| chr10 | 2241395 | C | T | Os10g0136150 | CDS | 0.586 | 0.975 |
| chr10 | 3516642 | C | T | Os10g0155800 | CDS | 0.533 | 0.650 |
| chr11 | 4445678 | C | G | Os11g0191300 | CDS | 0.552 | 0.688 |
| chr11 | 4620955 | G | A | Os11g0187500 | 3UTR | 0.849 | 0.873 |
| chr11 | 27650691 | G | A | Os11g0683500 | 3'UTR | 0.542 | 0.571 |

AFD1 refers to the AFD between *japonica* rice and northeastern weedy rice samples, AFD2 refers to the AFD between northeastern and eastern weedy rice samples.

**Supplementary Table 3.** Primer sequences used in qRT-PCR analysis.

| Gene ID | Forward primer | Reverse primer |
| --- | --- | --- |
| Os03g0122600 | GAAGATAGCCAAGCGATGCC | TGCTGAATGGGTGGGTGC |
| Os10g0136150 | ATCTTTGCCTCCGTTTACTC | GCTGACATTATCATCCTCCCT |
| Os11g0191300 | ATCCCGTTTCCTGGGTTTG | TGTCCTGAGAAGTAGGTGGTT |
| Os10g0155800 | CAGCCTCTACTCCAACCAGC | CGAAGAAATCGGGCACCTC |
| Os08g0227200 | CTTGTGCTGGCGATGCG | CAATGCTGATAGGCTCTGTCCC |
| Os01g0328400* | ACCACTTCGACCGCCACTACT | ACGCCTAAGCCTGCTGGTT |

*Gene Os01g0328400 was the reference gene UBQ5 used in this study.

**Supplementary Table 4.** Summary information of 9 RNA-seq samples at different germination stages at 12℃.

| Sample | Clean Reads | Total Base Pairs | Mapping rate | Unique mapping rate | Expressed genes |
| --- | --- | --- | --- | --- | --- |
| HLJ_stage_I | 27,430,332 | 3,459,644,540 | 96.29% | 81.87% | 21637 |
| HLJ_stage_II | 27,231,115 | 3,545,872,114 | 92.40% | 84.46% | 21693 |
| HLJ_stage_III | 23,304,906 | 2,941,041,442 | 93.58% | 82.81% | 20589 |
| LN_stage_I | 25,688,722 | 3,254,565,475 | 96.03% | 71.50% | 19629 |
| LN_stage_II | 30,912,971 | 3,773,343,457 | 94.76% | 84.46% | 23053 |
| LN_stage_III | 25,371,586 | 3,270,692,156 | 95.49% | 81.69% | 17287 |
| JS_stage_I | 25,914,239 | 3,100,235,035 | 93.75% | 84.64% | 20166 |
| JS_stage_II | 26,235,296 | 3,321,252,315 | 95.96% | 71.54% | 23379 |
| JS_stage_III | 23,848,438 | 2,885,045,494 | 94.99% | 86.19% | 18709 |
